# Obstructive Pharyngo-Laryngeal Hematoma Complicating Oral Anticoagulation: Case Report

**DOI:** 10.22038/ijorl.2025.82416.3772

**Published:** 2025

**Authors:** Maissa Lajhouri, Houda Chahed, Rania Laajailia, Rihab Lahmer, Azza Mediouni, Najeh Beltaief

**Affiliations:** 1 *Department of Otorhinoloaryngology Head and Neck Surgery, La Rabta Hospital, Tunisia.*

**Keywords:** Airway obstruction, Dyspnea, Acenocoumarol, Drug interaction

## Abstract

**Introduction:**

Spontaneous pharyngo-laryngeal hematoma in patients receiving oral anticoagulants is rare and potentially fatal because of upper airway obstruction. Many risk factors can cause potential hemorrhagic complications, such as drug interactions.

**Case Report:**

We present the case of a 74-year-old man who was on oral anticoagulation by acenocoumarol and presented with acute laryngeal dyspnea. The final diagnosis of pharyngo-laryngeal hematoma secondary to hypercoagulation was made based on the clinical, biological, and radiological features. Therefore, conservative management was initiated. Drug interactions between anticoagulants and macrolides were the probable underlying causes of excessive anticoagulation.

**Conclusion:**

Pharyngo-laryngeal hematoma should be considered in patients receiving anticoagulant therapy with symptoms of upper airway obstruction. Drug interactions may be the underlying cause of hypercoagulation, so medications should be carefully prescribed to these patients.

## Introduction

Major hemorrhagic complications associated with oral anticoagulation can occur in approximately 2% to 4% of patients (1).

The gastrointestinal tract and central nervous system are the most commonly affected sites. Pharyngo-laryngeal hematoma is an uncommon and potentially life-threatening bleeding complication (1-3). We report the clinical case of a patient who presented with a pharyngo-laryngeal hematoma related to excessive anticoagulation, probably due to preceding macrolide treatment. Our objective was to highlight the importance of considering drug interactions when prescribing new medicines, especially in older individuals under anticoagulation.

## Case Report

**Fig 1:**
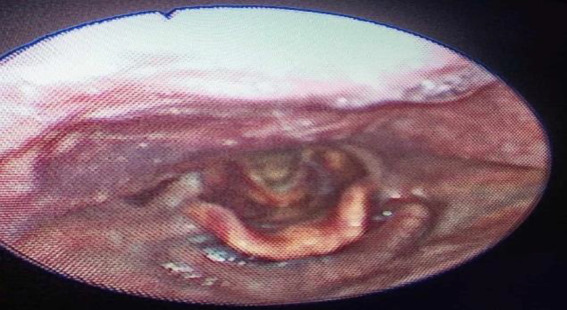
Pharyngo-laryngeal hematoma on fibrolaryn- goscopy

A 74-year-old man with a medical history of diabetes mellitus type 2, hypertension, ischemic heart disease, and atrial fibrillation on anticoagulation with acenocoumarol presented to the otorhinolaryngology emergency department with acute dyspnea, following a three-day history of dysphagia and voice changes. The cardiologist regularly followed up with the patient. He reported a tonsillitis episode treated with clarithromycin one week before the actual history. Physical examination revealed breathing difficulties. Laryngeal dyspnea was observed without any signs of respiratory struggle. The patient had a muffled voice. Vitals were stable, with normal temperature, regular cardiac rhythm at 80 beats per minute, normal arterial blood pressure, and oxygen saturation of 98% on room air. Cervical swelling without signs of inflammation was observed in the submental and submandibular regions. Oral cavity examination revealed hypersialorrhea with mucosal congestion on the floor of the mouth. Fibrolaryngoscopic examination revealed a bulging violaceous aspect of the posterior pharyngeal wall, epiglottis, aryepiglottic folds, and ventricular bands. The vocal cord folds were hemorrhagic, with normal movement (Figure 1). 

**Fig 2:**
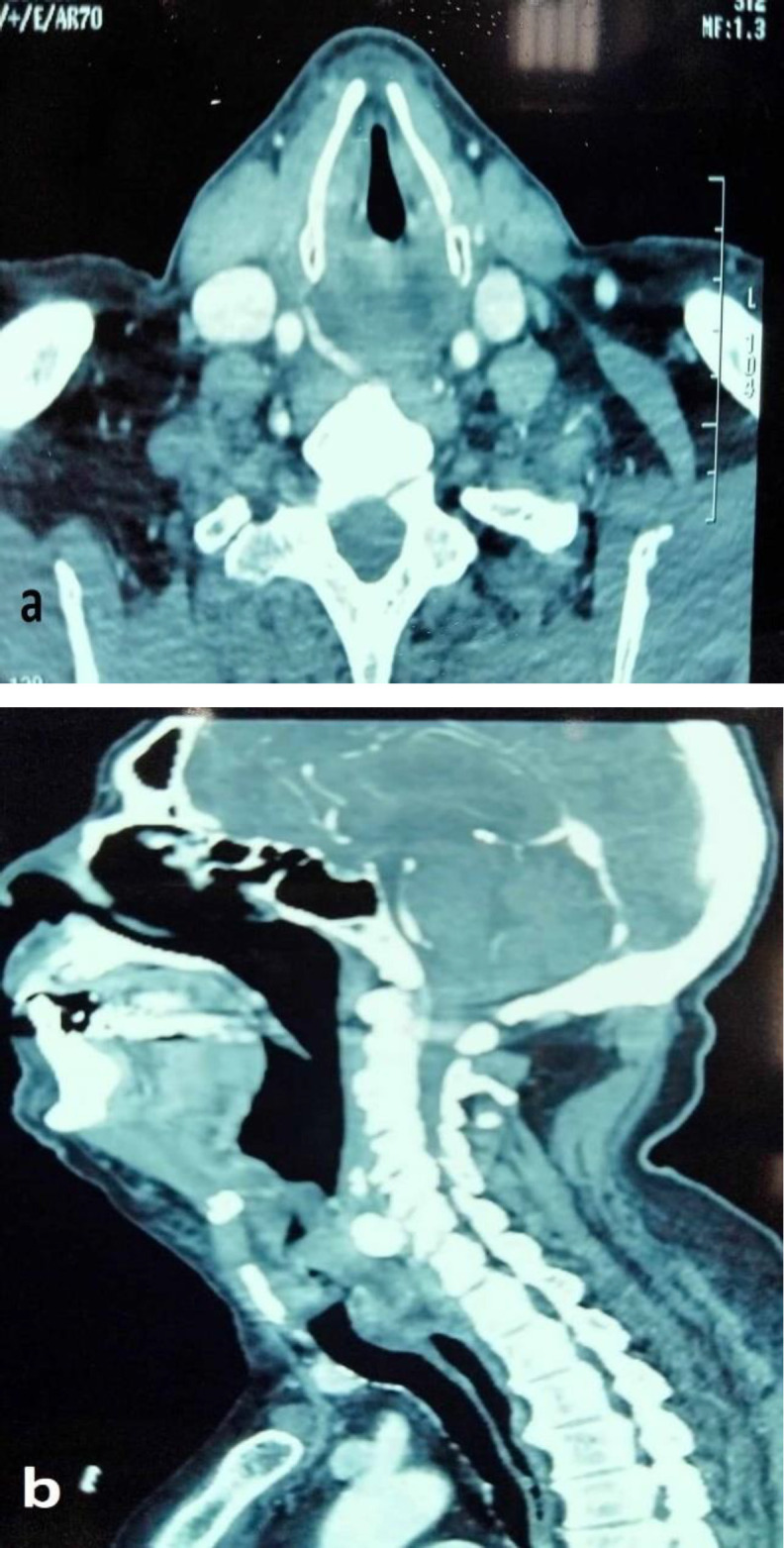
Paryngo-laryngeal soft tissue swelling on axial (a) and coronal (b) CT scan

Laboratory studies revealed a white blood cell count of 12000/mm^3^, hemoglobin of 15 g/dl, hematocrit of 44%, and platelet count of 383000. The prothrombin time was 90 s, with an elevated international normalized ratio (INR) of 7. Head, neck, and chest computed tomography (CT) revealed soft tissue swelling in the supraglottic, retropharyngeal, and submandibular regions (Figure 2). The lower airways and lung parenchyma were normal. 

The final diagnosis was pharyngo-laryngeal hematoma secondary to excessive anticoagulation. The patient was closely monitored. Acenocoumarol was discontinued, and the patient received intravenous Vitamin K; fresh frozen plasma was unavailable. Antibiotics (amoxicillin clavulanic acid) and corticosteroids were administered. 

The patient remained stable, with progressive improvement in dyspnea.

**Fig 3:**
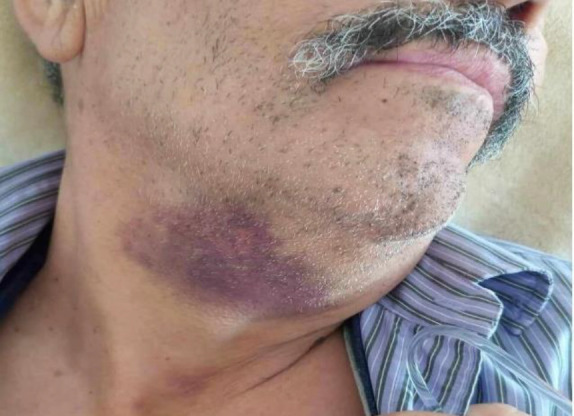
Subcutaneous ecchymosis in the anterior neck

On the second day after admission, ecchymosis of the mucosa of the floor of the mouth and cutaneous submandibular ecchymosis were observed (Figure 3). 

In the following days, an endoscopic examination revealed progressive hematoma resolution. Cardiologists have indicated the reintroduction of acenocoumarol at the usual dose. The patient was discharged when the therapeutic INR levels were obtained. He was informed of the need for regular follow-ups with his cardiologist and the necessity for careful prescription of acenocoumarol.

At the last follow-up, 9 months after admission, the patient was well, and the fibrolaryngoscopic examination was normal.

## Discussion

Pharyngo-laryngeal hematoma should be considered in patients on oral anticoagulation who present with pharyngo-laryngeal symptoms, such as dysphagia, dyspnea, and hoarseness. This complication has the potential for fatal outcomes owing to airway obstruction (3). In our patient, the diagnosis was based on clinical and biological features. Fibrolaryngos- copy confirmed this diagnosis.

Capps et al. reported a triad of symptoms associated with cervical-mediastinal hematoma: clinical signs of upper airway and pharyngoesophageal compression, anterior tracheal displacement on lateral cervical radiography, and secondary subcutaneous ecchymosis in the anterior neck and upper thorax (1,4,5). CT can be performed to reveal the bleeding extent (4,5). Therapeutic measures for over-anticoagulation include withholding anticoagulants, parenteral vitamin K, and fresh frozen plasma (3,4). Vitamin K allows the production of new coagulation factors that increase within 2 h and are normalized within 24 h. Fresh frozen plasma provides immediate vitamin K-dependent coagulation factors (4). Systemic steroids and antibiotics are controversial, although they have been used in several case reports (4-6).

Mild subjective respiratory symptoms led us to opt for conservative management. Airway management in patients with airway obstruction symptoms should be performed on an individual case basis (4,5,7,8). Rosenbaum recommended close supervision with airway manipulation only if necessary (4,8). Spontaneous anticoagulation-induced hemorrhage is associated with several risk factors that may cause hypercoagulation, such as diet (changes in dietary fat and vitamin K deficiency), medical conditions (hepatic dysfunction and hyperthyroidism), and drug interactions (4,7,9). Our patient developed a spontaneous hematoma secondary to a rare interaction between acenocoumarol and clarithromycin. Macrolides inhibit cytochrome P450, an enzyme involved in metabolizing many drugs, including anticoagulants (10). 

Grau reported a case of a drug interaction between clarithromycin and acenocoumarol in a 75-year-old woman. They demonstrated that clarithromycin may increase the anticoagulant effects of oral anticoagulants. Therefore, the combination should be avoided. If necessary, the anticoagulation effects should be frequently monitored (10).

## Conclusion

Although rare, pharyngo-laryngeal hematoma must be considered in patients on anticoagulation therapy who present with upper aerodigestive tract symptoms. Early diagnosis should be performed to permit conservative management. 

Drug interactions may be the underlying cause of hypercoagulation. Medications should be carefully prescribed, especially to patients receiving anticoagulant treatment. 

## References

[1] Chin KW, Sercarz JA, Wang MB, Andrews R (1998). Spontaneous cervical hemorrhage with near-complete airway obstruction. Head Neck..

[2] Kerr HD, Kwaselow A (1984). Vocal cord hematomas complicating anticoagulant therapy. Ann Emerg Med..

[3] Divya GM, Dahiya V, Ramachandran K, Muhammed F (2015). Drug-induced airway hematoma. Int J Otolaryngol Head Neck Surg..

[4] Bloom DC, Haegen T, Keefe M (2002). Anticoagulation and spontaneous retropharyngeal hematoma. J Emerg Med..

[5] Kabagenyi F, Hidour R (2022). Spontaneous retropharyngeal haematoma causing upper airway obstruction in a child: case report. Int Med Case Rep J..

[6] Ashraf A, Bannon M, Smith C, Kaushik P, Marak C (2022). Upper airway hematoma! An unusual presentation of acute upper airway obstruction. Respir Med Case Rep..

[7] Triaridis S, Tsiropoulos G, Rachovitsas D, Psillas G, Vital V (2009). Spontaneous haematoma of the pharynx due to a rare drug interaction. Hippokratia..

[8] Thomas SA, Moideen SP, George SM, Ipe S, Menon DG (2022). Management of spontaneous neck hematoma in patients on anticoagulant therapy: our experience from a tertiary care center. Indian J Otolaryngol Head Neck Surg..

[9] Olandsobo AG, Lazraq M, Bensaid A, Miloudi Y, El Harrar N (2018). Hématome cervical spontané et du plancher buccal : localisation inhabituelle d’un accident hémorragique aux antivitamines K. À propos d’un cas.

[10] Grau E (1996). Interaction between clarithromycin and anticoagulants. Ann Pharmacother..

